# Editorial: Engineering Rumen Metabolic Pathways: Where We Are, and Where Are We Heading

**DOI:** 10.3389/fmicb.2017.02627

**Published:** 2018-01-04

**Authors:** Emilio M. Ungerfeld, C. Jamie Newbold

**Affiliations:** ^1^Coordinación de Sistemas Ganaderos, Instituto de Investigaciones Agropecuarias INIA Carillanca, Temuco, Chile; ^2^Institute of Biological, Environmental and Rural Sciences, Aberystwyth University, Aberystwyth, United Kingdom

**Keywords:** rumen, pathways, metabolism, engineering, ruminants, microbial community, fermentation

The rumen plays a central role in the ability of ruminants to produce human edible food from resources that are otherwise not available for consumption by mankind. Fermentation in the rumen also has the potential to influence the health and well-being of both the host and humans through the nutritional quality of meat and milk, and the environment through potential deleterious environmental consequences of emissions of greenhouse gases and excessive nitrogen excretion in feces and urine. Given the importance of the rumen fermentation, it is perhaps not surprising that a great deal of effort has been devoted to investigating methods for manipulating this complex ecosystem, and the possibility of engineering rumen metabolic pathways is a highly attractive target.

By “Engineering” we do not refer specifically to genetic engineering; by “Engineering rumen metabolic pathways” we understand the manipulation of one or more rumen microbial processes toward the optimization of ruminant production and sustainability. As such, this e-book includes articles related to rumen fermentation, energy metabolism, and methane production. We have also included articles of microbial digestion and growth, on the understanding that these processes also impact ruminant production efficiency and sustainability. The e-book includes articles that report or review interventions to manipulate the rumen microbial community, as well as articles on basic experimental and theoretical research that help understanding the rumen microbial community toward its manipulation. The e-book compiled does not intend to be a comprehensive summarization of current knowledge or an update of all recent discoveries. It is proposed as a useful, rather than as a thorough, compilation of recent experimental and theoretical research on rumen microbiology and biochemistry.

An excellent overview of rumen microbial ecology is provided by Weimer. Weimer discusses how the rumen microbial community, while being complex and diverse, is remarkably stable due to its ecological redundancy in the role and physiology of microbial groups and the resilience to resist and recover from perturbations. Whereas these properties provide nutritional stability to the rumen and host, they also imply great challenges to engineer rumen function. Interestingly, recent research discussed by Yáñez-Ruiz et al. on how microbial colonization of the rumen soon after birth can be influenced by various factors, offers hope that early in the animals' life the rumen may be reprogramed in a somewhat stable way.

Protozoa are a very important group of microorganisms in the rumen microbial ecosystem. Newbold et al. review current knowledge about rumen protozoa and critically discuss their role in the microbial community and implications to the host's nutrition. They conclude that elimination of the ciliate protozoa increases microbial protein supply by up to 30% and reduces methane production by up to 11% whilst noting that as yet no method to control protozoa in the rumen that is safe and practically applicable has been developed.

Microbial protein synthesized in the rumen is the main and cheapest source of amino acids for ruminants. Hackmann and Firkins discuss in depth how energetically inefficient processes such as glycogen cycling and interconversion of volatile fatty acids can potentially impact microbial protein synthesis. Knowledge about these processes can allow designing strategies to minimize them and help optimizing microbial protein supply to the ruminant.

Soliva et al. investigated the efficiency of microbial synthesis from a nutritional viewpoint, revisiting the concept that there is a constant minimum requirements for rumen-degradable protein to support microbial digestion of organic matter across different feeds. Their study suggests that with tropical forages the required ratio of rumen degradable protein: apparently degraded organic matter might not be constant across high-fiber diets and that thresholds of either rumen degradable protein or ruminal ammonia concentration in existing models of rumen function may not be reflected appropriately by constants.

Microbial growth and protein synthesis requires utilization of ATP, which needs to be generated in fermentation. Efficiency and rate of ATP generation can thus impact microbial protein production. Hackmann and Firkins identified in most species of the genera *Butyrivibrio* and *Pseudobutyrivibrio* genes encoding for enzymes that acting in concert could allow for ATP generation through transmembrane electrochemical gradients. They propose the novel concepts that butyrivibrios could drive ATP generation by electron transport phosphorylation and that this energy conservation system might enhance the butyrivibrios' ability to overcome growth inhibition by unsaturated fatty acids.

Unsurprisingly given the current focus on greenhouse gas emissions from ruminant agriculture, methane production in the rumen, and its mitigation, provides a significant focus to our research topic. St-Pierre et al. review available information on the identification and occurrence of methanogenic archaea in the rumen and other gastrointestinal environments in herbivores, and speculate on how in the future such knowledge might lead to mitigation strategies targeting methanogens in the rumen. Latham et al. review the utilization of nitrate and nitrocompounds as methane-controlling strategies, as alternative electron acceptors that incorporate electrons at the expense of methane production and as direct inhibitors of methanogenesis. Despite demonstrated *in vitro* and *in vivo* efficacy of nitrate in mitigating rumen methane production, concerns remain over potential toxicity to the animal and increases in the emissions of nitrous oxide to the atmosphere, and further studies are required to quantify the risk versus the benefits as a practical approach in the field. Nitrocompounds, and more recently nitrooxycompounds, have been shown to effectively decrease methane production *in vitro* and *in vivo* in the long term, although as yet production benefits are still to be realized and the problem of accumulation of dihydrogen typically occurring along the inhibition of methanogenesis remains unresolved.

Whilst a decrease in methane emissions is expected to improve ruminant performance by decreasing energy losses as methane, decreases in methane production do not consistently result in greater performance. Benefits in energy efficiency caused by inhibiting methane production depend on the resulting alterations occurring in the flows of metabolic hydrogen. Changes in metabolic hydrogen flows as a consequence of inhibiting methane production have not been measured *in vivo* simultaneously with production of gases, but metabolic hydrogen balances can be calculated for published *in vitro* methanogenesis-inhibition experiments. Ungerfeld meta-analyzed shifts in metabolic hydrogen flows in 28 batch culture experiments and 16 continuous culture experiments in which methanogenesis was inhibited. He concluded that inhibiting methane production resulted in a moderate re-direction of reducing equivalents toward propionate in batch culture, but not in continuous culture. There was no benefit in heat of combustion in total volatile fatty acids by inhibiting methanogenesis, and a consistent decrease in metabolic hydrogen recovery. Guyader et al. further investigated the re-direction of metabolic hydrogen away from methane using three known methanogenesis inhibitors, nitrate, 3-nitrooxypropanol, and anthraquinone. Methane production was decreased by up to 75%, but despite increases in reduced volatile fatty acids production, recovery of metabolic hydrogen was still considerably lower than 100%, highlighting the need to identify and study unknown metabolic hydrogen sinks in the rumen.

Engineering the flows of metabolic hydrogen in the rumen requires understanding how these flows are physicochemically controlled. Through estimating the thermodynamic feasibility of the incorporation of accumulated dihydrogen into propionate and butyrate and reductive acetogenesis, Ungerfeld attempted to understand why dihydrogen generally accumulates when methanogenesis is inhibited. In that regard, insightful experiments by Denman et al., Martínez-Fernández et al., and Martínez-Fernández et al. examined the changes in the rumen microbiome occurring during methanogenesis inhibition. Denman et al. showed that inhibiting methanogenesis in goats stimulated the abundance of reads of enzymes from the propionate randomizing pathway, in agreement with increases in the abundance of microbial groups producing propionate. In contrast, inhibiting methanogenesis did not stimulate reductive acetogenesis. Martínez-Fernández et al. conducted metabolomic analyses that revealed an increase in amino acids, organic and nucleic acids in rumen fluid with inhibition of methanogenesis. Importantly, the main known fibrolytic bacteria, as well as protozoa and fungi, seemed mostly unaffected by inhibition of methanogenesis. Martínez-Fernández et al. successfully decreased accumulated dihydrogen in the methanogenesis-inhibited rumens of steers through the supplementation of phloroglucinol, a metabolic intermediate of flavonoids in the rumen.

Almost all measurement of dihydrogen concentration in rumen fermentation *in vitro* and *in vivo* have been conducted in the gas phase. Equilibrium between gaseous and dissolved dihydrogen has then been assumed to estimate the concentration of dissolved dihydrogen to calculate the thermodynamic feasibility of rumen pathways producing or incorporating dihydrogen. For the first time, Wang et al. simultaneously determined the concentration of dihydrogen in the gas and fluid phases to discover that they were not at equilibrium, and that dissolved dihydrogen was supersaturated in the rumen of sheep. This has important implications for the understanding of rumen thermodynamics.

The rate of digestion of a substrate influences the profile of fermentation products. Two studies in this e-book have implications in the understanding of the kinetics of fiber digestion. Griffith et al. found responses in digestibility of barley straw in semi-continuous culture to fast-digesting inoculum when barley straw was ammonia fiber expansion treated, but no differences between fast and slow-digesting inocula in untreated barley straw. Oss et al. investigated the effect of inocula from cattle and bison and their mixtures on fermentation in semi-continuous cultures, finding that bison inoculum increased fiber and protein digestibility. Both studies confirm the role of the microbial community inoculated in semi-continuous cultures in determining the capacity to digest fiber. Although a challenging objective, the rate or the extent of fiber digestion or both might respond to the manipulation of the microbial community.

Complexities of the rumen acidosis phenomenon are unraveled in the research by Plaizier et al. These authors compared the impact on the rumen, cecal, and fecal bacterial communities during subacute acidosis induced by two contrasting diets in which part of the forage was replaced by either rapidly degradable starch pellets or pellets of ground alfalfa. Different changes in microbial communities induced by the different acidotic challenges confirm that our understanding of the acidotic rumen is still incomplete. A better understanding of the different ways the acidosis phenomenon occurs and the microbial populations involved may allow differentially manipulating key microbial groups to control different types of acidosis.

Bannink et al. present a thorough analysis of how mechanistic mathematical modeling can help understanding digestion, fermentation, absorption of volatile fatty acids, and the control of rumen pH. The authors also explain the contribution of dynamic modeling to predict rumen nitrogen balance and how prediction of the volatile fatty acids profile can be aided by recent developments in the characterization of the rumen microbiome.

Microbial digestion and fermentation in the rumen provides ruminants with the flexibility to use fiber and non-protein nitrogen unavailable to humans and other animals, but at the same time results in energy losses as methane, and often inefficient utilization of nitrogen, both of which have negative consequences for the environment. Making ruminant production more efficient and sustainable will ineludibly involve manipulating rumen microbial activity. Unlike the fragility of other ecosystems, the redundancy and resilience of the rumen microbial community makes it a challenging target to manipulate. Whilst currently metabolic engineering of the rumen to achieve theoretical potentials is not yet possible, we continue approaching that goal as we develop our understanding of rumen microbiology and biochemistry. Some main roadblocks to be tackled are digestion, metabolic hydrogen management, nitrogen and fatty acids metabolism, and acidosis. These areas should not be seen as separate compartments, and the existence of interphases can result in interventions on one aspect having consequences on others. We foresee future developments resulting from the integration of microbial ecology multiomics techniques, in particular regarding the expression of functional genes, with the application of physical-chemistry principles and the refinement of thermodynamic and kinetic measurements in the rumen environment.

## Author contributions

All authors listed have made a substantial, direct and intellectual contribution to the work, and approved it for publication.

### Conflict of interest statement

The authors declare that the research was conducted in the absence of any commercial or financial relationships that could be construed as a potential conflict of interest.

